# Organ function support in patients with coronavirus disease 2019: Tongji experience

**DOI:** 10.1007/s11684-020-0774-9

**Published:** 2020-05-14

**Authors:** Yong Li, Fan He, Ning Zhou, Jia Wei, Zeyang Ding, Luyun Wang, Peng Chen, Shuiming Guo, Binhao Zhang, Xiaoning Wan, Wei Zhu, Multidisciplinary Team for COVID-19

**Affiliations:** 1 Department of Respiratory and Critical Care Medicine, Department of Internal Medicine, Tongji Hospital, Tongji Medical College, Huazhong University of Science and Technology, Wuhan, 430030 China; 2 Department of Nephrology, Department of Internal Medicine, Tongji Hospital, Tongji Medical College, Huazhong University of Science and Technology, Wuhan, 430030 China; 3 Department of Cardiology, Department of Internal Medicine and Genetic Diagnosis Center, Tongji Hospital, Tongji Medical College, Huazhong University of Science and Technology, Wuhan, 430030 China; 4 Hubei Key Laboratory of Genetics and Molecular Mechanism of Cardiological Disorders, Tongji Hospital, Tongji Medical College, Huazhong University of Science and Technology, Wuhan, 430030 China; 5 Department of Hematology, Tongji Hospital, Tongji Medical College, Huazhong University of Science and Technology, Wuhan, 430030 China; 6 Hepatic Surgery Center, Tongji Hospital, Tongji Medical College, Huazhong University of Science and Technology, Wuhan, 430030 China; 7 Hubei Key Laboratory of HPB Diseases, Tongji Hospital, Tongji Medical College, Huazhong University of Science and Technology, Wuhan, 430030 China; 8 Department of Emergency and Critical Care Medicine, Tongji Hospital, Tongji Medical College, Huazhong University of Science and Technology, Wuhan, 430030 China; 9 Optical Valley Branch of Tongji Hospital, Tongji Medical College, Huazhong University of Science and Technology, Wuhan, China

**Keywords:** COVID-19, severe and critical type, organ function support

## Abstract

Coronavirus disease 2019 (COVID-19) is a highly contagious disease and a serious threat to human health. COVID-19 can cause multiple organ dysfunction, such as respiratory and circulatory failure, liver and kidney injury, disseminated intravascular coagulation, and thromboembolism, and even death. The World Health Organization reports that the mortality rate of severe-type COVID-19 is over 50%. Currently, the number of severe cases worldwide has increased rapidly, but the experience in the treatment of infected patients is still limited. Given the lack of specific antiviral drugs, multi-organ function support treatment is important for patients with COVID-19. To improve the cure rate and reduce the mortality of patients with severe- and critical-type COVID-19, this paper summarizes the experience of organ function support in patients with severe- and critical-type COVID-19 in Optical Valley Branch of Tongji Hospital, Wuhan, China. This paper systematically summarizes the procedures of functional support therapies for multiple organs and systems, including respiratory, circulatory, renal, hepatic, and hematological systems, among patients with severe- and critical-type COVID-19. This paper provides a clinical reference and a new strategy for the optimal treatment of COVID-19 worldwide.

### Introduction

Since December 2019, a number of patients with novel coronavirus pneumonia have been found in Wuhan City, Hubei Province, China. As an acute respiratory infectious disease, it is classified under Class B infectious diseases as stipulated in the Law of the People's Republic of China on the Prevention and Control of Infectious Diseases and is managed as a Class A infectious disease. Aside from respiratory dysfunction, the disease also causes severe injuries to the circulatory, kidney, hepatic, and hematological systems and even leads to death [1,2]. Therefore, we mainly referred to the diagnosis and treatment protocol for COVID-19 (7th interim edition) [3] and other relevant guidelines and references, summarized the clinical experience, and compiled the organ dysfunction diagnosis and treatment plan of COVID-19.

## 1 Respiratory function support

### 1.1 Monitoring of laboratory index and special inspection

#### *1.1.1 Clinical indicators*

Regularly monitored and tested indicators included blood, urine routine test, C-reactive protein (CRP), procalcitonin (PCT), cytokines, liver function, myocardial enzymes, N-terminal pro-brain natriuretic peptide, renal function, lactic acid, electrolyte balance, coagulation function, lymphocyte subsets, blood gas, and imaging. Among these indicators, focus was given to peripheral blood lymphocyte counts, CRP, PCT, cytokines, D-dimers, lactic acid, electrolytes, and blood gas. For patients with critical-type COVID-19, the laboratory indexes mentioned above should be monitored daily.

#### *1.1.2 Cytokines*

Cytokines are important indicators for evaluating the severity of COVID-19. The levels of interleukin-2 receptor (IL-2R) and interleukin-6 (IL-6) in patients with severe- and critical-type COVID-19 are remarkably higher than those in patients with mild-type COVID-19. The IL-2R levels of patients with severe- and critical-type COVID-19 are 902 $\pm$ 51 U/mL and 1185 $\pm$ 80 U/mL, respectively, and their IL-6 levels are 52 $\pm$ 11 ng/L and 108 $\pm$ 12 ng/L, respectively [4]. Cytokine levels in the above range could induce inflammatory storms and aggravate the condition of patients in the short term. Cytokine changes should be monitored dynamically, and early intervention, such as glucocorticoid treatment and continuous renal replacement therapy (CRRT), should be performed.

### 1.2 Identification of patients with severe- and critical-type COVID-19

#### *1.2.1 Criteria of severe-type COVID-19*

(1) Respiratory frequency (RR) $\geqslant$ 30 times/min.

(2) Finger pulse oxygen saturation $\leqslant$ 93% in resting state.

(3) Oxygenation index ($PaO_2/FiO_2$) $\leqslant$ 300 mmHg (1 mmHg = 0.133 kPa), and the altitude should be corrected according to the following formula: $PaO_2/FiO_2$ $\times$ (atmospheric pressure (mmHg) /760).

(4) Pulmonary imaging shows significant progression of lesions > 50% within 24–48 h.

(5) Continuously high fever ( > 38.5 °C).

(6) Patients with COVID-19 aged $\geqslant$ 65 years or $\geqslant$ 50 years with high neutrophils, low lymphocytes, and neutrophil-to-lymphocyte ratio (NLR) $\geqslant$ 3.13 [5–7].

(7) Severe chronic underlying diseases: hypertension, diabetes, coronary heart disease, malignant tumors, structural lung disease, pulmonary heart disease, and immune-suppression.

#### *1.2.2 Criteria of critical-type COVID-19*

(1) Respiratory failure with mechanical ventilation.

(2) Shock.

(3) Combined with other organ failures and requiring intensive care unit (ICU) monitoring and treatment [3,6,7].

Evaluation of the severity of condition should not only be based on a single clinical indicator. It should be comprehensively assessed according to the patient's symptoms, peripheral blood lymphocytes, lactic acid, blood oxygen saturation, arterial blood gas, imaging, cytokines, and other indicators. For critical patients, early intervention, including non-invasive and invasive mechanical ventilation and glucocorticoid, should be considered to provide respiratory assistance and reduce inflammatory storms and exudation. Maintenance of internal environment balance and protection of multi-organ functions are also important.

### 1.3 Antiviral and bacterial treatment

#### *1.3.1 Antiviral treatment*

Antiviral drugs are as follows:

(1) α-interferon (5 million U or equivalent per adult, add 2 mL of sterile water for injection, aerosol inhalation 2 times a day).

(2) Lopinavir/ritonavir ((200 mg lopinavir + 50 mg ritonavir)/capsule, 2 capsules each time, 2 times a day, the course of treatment should not exceed 10 days).

(3) Chloroquine phosphate (18–65-year-old adults: for patients who weigh more than 50 kg, 500 mg each time, twice daily for 7 days; for patients who weigh less than 50 kg, 500 mg each time on the first and second days, twice daily, 500 mg each time on the third to seventh days, once a day).

(4) Arbidol (200 mg for adults, 3 times a day, the course of treatment should not exceed 10 days).

Chloroquine phosphate should be avoided in patients with heart disease. The interaction between chloroquine and quinolone drugs should be considered. Blood routine (white blood cells, hemoglobin), electrocardiogram (QT interval), vision, and mental and psychological conditions should be monitored regularly every 2–3 days during the chloroquine treatment. The toxicity of arbidol to the heart should be monitored.

Currently, antiviral drugs are effective for some patients with COVID-19. Thus, antiviral drugs should be used with caution. The use of three or more antiviral drugs at one time is not recommended. When intolerable side effects occur, the antiviral drugs should be stopped. For pregnant patients, gestational weeks must be considered, drugs with few side effects should be used, and the treatment risk must be explained.

*1.3.2 Antibacterial treatment*

Due to the gastrointestinal disorders of quinolone treatment and interactions with other antiviral drugs, second- and third-generation cephalosporins could be preferred for patients with bacterial infection.

(1) Patients' symptoms, blood routine test, CRP, PCT, imaging, and airway secretion culture can be used for comprehensive evaluation with or without antibacterial treatment.

(2) For patients with low lymphocyte count and treatment course longer than 2 weeks, the use of antibacterial drugs should be based on white blood cell (WBC) count, neutrophil percentage, and pulmonary imaging instead of PCT and CRP.

(3) For patients who accepted glucocorticoid treatment, invasive mechanical ventilation or extracorporeal membrane oxygenation (ECMO) support, and long-term use of broad-spectrum antibacterial drugs, the possibility of combined fungal infections should be considered and BG-β-D-glucan(1-3)-β-D/galactomannan (G/GM) test and culture of airway secretions should be performed [6,7].

### 1.4 Anti-mucus and anti-fibrotic treatment

Pulmonary edema and hyaline membrane formation were observed in pathological samples of COVID-19. Consolidation, local hemorrhage and necrosis, and even hemorrhagic infarction were observed in lung tissue. Serum, fibrinous exudate, and transparent membrane formation were observed in the alveolar cavity; part of the alveolar cavity exudate was mechanized, and even interstitial fibrosis was observed. A large number of sticky secretions were observed in the airway, part of the epithelium of the bronchial mucosa had fallen off, and mucus and mucus plugs were observed in the bronchus [3].

In consideration of the pathological features of pulmonary edema, hyaline membrane formation, interstitial fibrosis, and mucus plug formation in the airway, glucocorticoid and high-dose hydrochloride should be considered for severe- and critical-type patients to reduce inflammatory exudation and mucus formation, respectively. Acetylcysteine and alveolar surfactants should also be used to resist interstitial fibrosis, keep alveoli stable, and prevent atelectasis, respectively.

Specific usage is recommended as follows:

(1) Ambroxol hydrochloride, 300 mg for adults, 3 times a day, intravenous drip (i.v. drip).

(2) N-Acetylcysteine tablets 600 mg, 2–3 times a day, orally administered.

(3) 70 mg of bovine alveolar surfactant prepared with 2 mL sterilized water for endo-tracheal drip, 3 times a day.

### 1.5 Application of glucocorticoid

The use of glucocorticoid is currently debatable. Glucocorticoid therapy should be given at an appropriate dose and course at the right time.

*1.5.1 Master the timing of glucocorticoid therapy*

COVID-19 development usually occurs within 7–10 days, which usually manifests as uncontrollable high fever, exacerbation of dyspnea, progressive deterioration of oxygenation index, rapid progress of imaging, and sharp increase of cytokines after treatment, indicating over-activated inflammatory response and cytokine storm. For such patients, early intervention of glucocorticoid is recommended.

*1.5.2 Dose and course of treatment*

The recommended dose of methylprednisolone is 1–2 mg/kg/day. In general, 40 mg methylprednisolone should be taken once a day or two times a day. If high fever cannot be controlled, dyspnea cannot be improved, and indicators are progressively deteriorated, the dose of glucocorticoid needs to be appropriately increased. The daily dosage of methylprednisolone should be gradually reduced after 3–5 days. The treatment course is 5–7 days [8]. The dosage and course of glucocorticoid used in some critical-type patients need to be adjusted according to actual conditions. The adverse of glucocorticoid should be closely monitored.

*1.5.3 Prevention of infection*

For patients treated with glucocorticoid, third-generation cephalosporins can be used to prevent infection.

*1.5.4 Use glucocorticoid with caution*

(1) Patients with diabetes are receiving oral medication or insulin treatment.

(2) Allergy to known methylprednisolone, hydrocortisone, dexamethasone, or other excipients.

(3) Refractory hypertension.

(4) Epilepsy or delirium.

(5) Glaucoma.

(6) Known active gastrointestinal bleeding in the past 3 months.

(7) Known hypokalemia that is difficult to correct.

(8) Known secondary bacterial or fungal infections.

(9) Known immunosuppressive status (such as chemotherapy/radiotherapy/HIV infection within one month after surgery).

(10) Severe lymphopenia (the absolute value of peripheral blood lymphocytes is less than 300/μL) [8].

### 1.6 Respiratory support therapy

#### *1.6.1 Oxygen therapy*

Including nasal catheter and mask oxygen, for hypoxemia ($PaO_2$ < 60 mmHg or $SpO_2$ < 93%), respiratory distress (breathing frequency > 24 times/min), and hypotension (systolic blood pressure) < 100 mmHg), oxygen therapy should be considered. The initial oxygen flow rate should be set to 5 L/min, and $SpO_2$ should maintain at 94%–98% (at least 95% for pregnant patients) by adjusting the oxygen flow rate. Patients with chronic hypercapnia, such as chronic obstructive pulmonary disease, should maintain $SpO_2$ at 88%–92% [8]. Conditions of respiratory distress and hypoxemia should be assessed by blood oxygen saturation and arterial blood gas. Other respiratory support methods should be used in the absence of significant improvement.

#### *1.6.2 Non-invasive ventilation (NIV) and high-flow nasal cannula (HFNC) oxygen therapy*

NIV or HFNC can be considered in patients with respiratory distress and/or hypoxemia that cannot be relieved after receiving standard oxygen therapy.

(1) NIV

Pathophysiological changes in the lungs of patients with critical-type COVID-19 not only show acute respiratory distress syndrome (ARDS)-related ventilation/perfusion defects but also diffuse dysfunction due to pulmonary interstitial disease and fibrosis. Therefore, oxygen saturation remains poor in some patients after tracheal intubation and invasive mechanical ventilation. Inability of weaning from mechanical ventilation also leads to complications, such as ventilator-associated pneumonia, in the later weeks of treatment. The mortality of patients with invasive mechanical ventilation remains high because of the abovementioned factors. NIV has the advantage of positive pressure, and positive end-expiratory pressure (PEEP) can prevent partial alveolar collapse and improve ventilation/perfusion defects. Therefore, we recommend NIV assistance for patients with severe tendencies who can tolerate NIV. The initial parameters could set to IPAP 8–10 $cmH_2O$, EPAP 5–8 $cmH_2O$, and $FiO_2$ 100%. If the patient cannot tolerate that situation at the initial stage, adjustments should be made in terms of nasal mask selection, sealing, parameters, and so on. The observation time of using a NIV is generally 2 h (dynamic adjustment parameters during the period according to the patient's condition, tidal volume (Vt), and blood oxygen saturation). If this situation of Vt ⩽ 9 mL/kg, RR ⩽ 30 times/min, $PaO_2/FiO_2$ can be maintained, NIV may continue; if Vt 9–12 mL/kg, $PaO_2/FiO_2$ is stable, close monitoring should be performed for 6 h, if Vt > 12 mL/kg or $PaO_2/FiO_2$ gets worse, NIV should be stopped and replaced with tracheal intubation for invasive mechanical ventilation.

(2) HFNC treatment

HFNC treatment can be selected for the following conditions: mild to moderate hypoxemia (100 mmHg ⩽ $PaO_2/FiO_2$ < 300 mmHg), no indication of emergency tracheal intubation, and relatively stable conditions. Patients with mild ventilation dysfunction (pH ⩾ 7.3) should be used with caution but should be prepared to switch to noninvasive or invasive positive pressure ventilation at any time. Hemodynamic instability, multiple organ failure, or unconsciousness should avoid using HFNC. HFNC gas flow can be adjusted to a maximum of 60–70 L/min, and $FiO_2$ can be adjusted from 0.21 to 1.0. Parameter setting: (i) Type I respiratory failure: the gas flow (Flow) is initially set to 30–40 L/min and the flow is gradually increased to 50–60 L/min after the patient tolerates that; $FiO_2$ is adjusted to maintain pulse oxygen saturation ($SpO_2$) at 92%–96%, combined with dynamic adjustment of blood gas analysis; if the oxygenation target is not reached, the $FiO_2$ can be gradually increased and adjusted up to 1.0; the temperature setting range is 31–37 °C, depending on the patient's comfort and tolerance; (ii) Type II respiratory failure: HFNC should be used with caution in patients with COVID-19 with type II respiratory failure. The initial setting of the gas flow (Flow) is 20–30 L/min, which is adjusted according to the patient's tolerance and compliance; if the patient has obvious carbon dioxide retention, the flow can be set at 45–55 L/min or higher if the patient can tolerate that; $FiO_2$ is titrated to maintain $SpO_2$ at 88%–92%, and it is dynamically adjusted in conjunction with blood gas analysis; the temperature setting range is 31–37 °C, which is adjusted appropriately according to the patient's comfort and tolerance, and the sputum viscosity [6,7,9].

The criteria of NIV and HFNC oxygen therapy failure are as follows.

(1) Finger pulse oxygen saturation ⩽ 90% and/or RR ⩾ 30 times/min.

(2) Hypercapnia, respiratory acidosis (pH ⩽ 7.25).

(3) Hemodynamic instability.

(4) Multiple organ failure.

(5) Disorders of consciousness.

(6) Patients are extremely uncooperative.

*1.6.3 Invasive mechanical ventilation*

It should be converted to invasive mechanical ventilation in time when NIV and HFNC are ineffective. We would like to propose to extend the standard of oxygenation index for mechanical ventilation.

Lung protective ventilation should be adopted. It is recommended to use small tidal volume (4–8 mL/kg (ideal body weight)) and low plateau pressure ventilation ( $< 30$ $cmH_2O$).

(1) Setting of mechanical ventilation parameters: The initial tidal volume (Vt) can be set to 6 mL/kg. If the ventilation is insufficient, the Vt can be appropriately increased to 8 mL/kg. The "permissible hypercapnia" strategy can be used to reduce the Vt as much as possible and ensure that the pH is $> 7.25$. After setting Vt, the pressure index must be monitored and the pressure of the inhalation platform must be controlled below 30 $cmH_2O$. When the airway platform pressure is $\leqslant 35$ $cmH_2O$, high PEEP can be used appropriately. Airway warming and humidification should be emphasized to avoid blood crust and mucus plug to block airways. If the platform pressure is $> 30$ $cmH_2O$ or the driving pressure is $> 15$ $cmH_2O$, Vt should be gradually reduced at a speed of 1mL/kg until the platform pressure $< 30$ $cmH_2O$ and the driving pressure $< 15$ $cmH_2O$ or Vt is reduced to 4 mL/kg (ideal body weight). At the same time, Vt is lowered and RR is increased to ensure alveolar minute ventilation and avoid $CO_2$ retention. For every 1 mL/kg Vt reduction, RR needs to be increased 5 times per minute. After increasing the RR, ventilator flow rate time curve should be observed. If the spontaneous breathing is too strong and the Vt is too large ( $> 8$ mL/kg), the sedative and analgesic dose can be increased and muscle relaxants can be used for a short time. If the $CO_2$ retention is still progressive, prone position ventilation (PPV) or ECMO is recommended [6,7,9].

(2) Mechanical ventilation in a prone position is mainly used to improve the patient's blood oxygen saturation. The principle is to effectively improve the ventilation/perfusion defects and make the dorsal collapsed alveoli expand. It can also make the lungs and tracheal secretions get a good drainage under the action of gravity and reduce the pressure of the heart and mediastinum on the sagging lung area. Therefore, it is beneficial for the drainage of sputum in the deep and small bronchial tubes of patients and for the control of lung infections. PPV is an important method for lung protection and lung re-expansion in patients with severe- and critical-type COVID-19. For patients with moderate-to-severe ARDS ($PaO_2/FiO_2 \leqslant 150$ mmHg), daily prone ventilation must be implemented for $> 12$ h as soon as possible [6,7,9].

We do not generally recommend strategy of lung expansion due to complications, such as barotrauma or pneumothorax.

*1.6.4 ECMO treatment*

ECMO is recommended for patients with severe- and critical-type COVID-19 with reversible etiology in the early stage. VV-ECMO is preferred for patients with simple respiratory failure, and VA-ECMO is used for circulatory failure. The mechanical ventilation setting during ECMO treatment is based on the principle of ultra-protective ventilation of the lungs, Vt is set to 2–4 mL/kg (ideal body weight), the platform pressure is controlled to $< 25$ $cmH_2O$, and RR is 8–10 times/min. In the absence of contraindication, PPV should be used in combination with ECMO treatment [6,7,9].

### 2 Circular function support

#### 2.1 Risk assessment of cardiovascular disease in patients with COVID-19

High risk of cardiovascular events should be considered in patients with COVID-19, if one of the following conditions was observed:

(1) Previous medical history showed organic heart disease (including coronary heart disease, cardiomyopathy, congenital heart disease, and heart failure caused by various reasons; the cardiovascular risk of diabetes should be considered the same as coronary heart disease).

(2) Patients with risk factors of cardiovascular diseases, such as obesity, hypertension, and hyperlipidemia.

(3) Patients with progressive increase of serum hypersensitive troponin (cTn) and N-terminal pro B type natriuretic peptide (NT-proBNP) after admission.

For all patients with high-risk cardiovascular events, electrocardiogram and echocardiography must be performed as soon as possible, and the trend of cTn and NT-proBNP should be monitored.

#### 2.2 Identification and treatment of fulminant myocarditis

Heart is an important target organ for the inflammatory storm caused by COVID-19. Fulminant myocarditis should be highly suspected when patients with COVID-19 have the following conditions:

(1) A significant increase in cTn that cannot be explained by hypoxemia. Unlike the mild to moderate increase in myocardial infarction, the increase of cTn in fulminant myocarditis is up to tens of thousands folds of the upper limit of normal range. The significant elevation of cTn is essential for the diagnosis of fulminant myocarditis, and a significant increase in NT-proBNP could be observed.

(2) Fulminant myocarditis should be highly suspected in the presence of hemodynamic instability or shock in patients with significant elevation of cTn. Electrocardiogram and echocardiography should be performed immediately to differentiate from acute myocardial infarction (AMI).

(3) If fulminant myocarditis is highly suspected, mechanical life supports such as intra-aortic balloon pump (IABP) or ECMO should be considered immediately under the assessment of cardiologist.

(4) According to Chinese Society of Cardiology expert consensus statement on the diagnosis and treatment of adult fulminant myocarditis [10], for patients with COVID-19 and fulminant myocarditis, immunological modulation (sufficient doses of glucocorticoid 200 mg q.d. i.v. and intravenous immunoglobulin 10–20 g q.d. i.v. drip) should be administered to mechanical life supports.

### 2.3 Management of COVID-19 patients with high risk of cardiovascular disease

#### *2.3.1 Blood pressure (BP) management*

(1) For the management of hypertension in patients with COVID-19, a BP target of less than 140/90 mmHg is recommended to reduce the afterload of the heart. No solid evidence shows that any kind of antihypertensive drugs increase the risk of COVID-19 infection in patients. If patients develop hypotension during treatment, antihypertensive pharmacotherapy should be discontinued immediately.

(2) If disease progression leads to hypotension, vasoactive agents (e.g., norepinephrine or dopamine) can be administered to maintain stable hemodynamics. Mechanical circulation support (e.g., IABP or ECMO) is preferred for patients with significantly elevated serum troponin (more than 10 times the upper limit of normal range). Drug therapy is still recommended to maintain BP at a relatively safe level (90/60 mmHg or above) when mechanical circulation support is unavailable. The dose of vasoactive drugs should be as low as possible. Vasoactive agents or positive inotropic drugs (e.g., cedilanid or digoxin) should not be excessively used in patients with severe myocardial injury because of their side effect of increased loading of the heart.

#### *2.3.2 Heart rate and rhythm management*

(1) Treatment of tachycardia

Sinus tachycardia is the most common arrhythmia in patients with COVID-19. Identification of the underlying cause of sinus tachycardia should be considered first, including anxiety, pain, hypoxia, effective hypovolemia, fever, infection, hyperthyroidism, ventilator, or insufficient sedation after invasive support. Sinus tachycardia is expected to be resolved with correction of the underlying cause. Recommendations include moderate sedation, analgesia, anxiolysis, strengthening anti-infection, and improving ventilation and oxygenation. If resting sinus heart rate remains above 100 bpm, ivabradine can be used. If rapid atrial fibrillation complicated with heart failure occurs, digitalis can be used orally (digoxin tablets 0.125 mg q.d.) or intravenously (cedilanid 0.2–0.4 mg i.v. bolus, reusable, up to 1.2 mg per 24 h). Blood potassium must be maintained at a normal level to prevent intoxication during digitalis administration. New-onset atrial fibrillation in patients with COVID-19 is usually induced by stress (e.g., infection), which belongs to paroxysmal atrial fibrillation. After electrolyte imbalance, fever, infection, and other incentives are ameliorated, most atrial fibrillation is terminated spontaneously. If atrial fibrillation lasts for 48 h, anticoagulation treatment should be performed (full dose of low-molecular-weight heparin subcutaneous injection is recommended).

(2) Treatment of bradycardia

No special treatment is indicated in the following cases: resting heart rate above 40 bpm while awake, normal ECG, hemodynamically stable, asymptomatic. For patients with hemodynamic instability, amaurosis, or even syncope, isoproterenol (1 mg isoproterenol + 50 mL normal saline intravenously pumped at an initial dose of 1–2 mL/h) can be used to increase the heart rate to 50–60 bpm. If drug effect is poor or drug-related arrhythmia occurs, temporary pacemaker implantation with a floating catheter should be considered.

#### *2.3.3 Liquid management*

The amount of liquid in and out of patients suffering from COVID-19 and complicated with high risk of cardiovascular events should be recorded in 24 h. Fluid management is an important aspect. The basic principle is to control liquid inflow strictly and avoid excessive liquid inflow or outflow in a short time.

#### *2.3.4 Electrolyte management*

The balance of electrolytes and acid–base, especially the concentration of $K^+$, must be monitored closely in patients with high risk of cardiovascular events. In the case of normal renal function or CRRT support, the concentration of potassium was recommended to be maintained at 4.0–5.0 mmol/L. Oral potassium tablets or potassium-containing solutions are effective treatments for supplementing potassium. If patients cannot take potassium chloride tablets orally, potassium chloride solution can be injected through the gastric tube. Preventive supplementation of potassium chloride solution 30–40 mL/day is extremely important for patients with poor diet. Intravenous supplementation should be considered for patients who are severely deficient in potassium but cannot supplement orally. The common method is to add 10 mL of 10% potassium chloride to 500 mL of 5% glucose solution. Intravenous infusion of potassium chloride must be slow, ensuring the infusion rate is 1 g potassium chloride every 30–40 min. Rapid intravenous injection is not advisable for supplementation of potassium because a sudden increase in the level of serum potassium may cause cardiac arrest. CRRT is useful in controlling the concentration of serum potassium at a safe level.

##### *2.3.5 Medication for chronic diseases*

The medication details of patients with chronic diseases, such as hypertension, should be collected after admission. Medications that patients took for a long time before admission, such as antihypertensive drugs, hypoglycemic drugs, antiplatelet drugs, lipid-lowering drugs, should be recorded. If the patient has recently undergone stent implantation or valve replacement, a consult of cardiovascular specialists should be requested for guiding treatment. Suddenly discontinuing the chronic drugs (such as antiplatelet drugs, anticoagulants, and antihypertensive drugs) may lead to aggravation of the original disease or even cause sudden death.

##### *2.3.6 Ameliorate myocardial metabolism*

Hypoxia is the most common manifestation of patients with COVID-19, especially in severely or critically ill patients. Thus, the myocardial metabolism in these patients should be highly valued. The therapy of improving myocardial metabolism should be implemented in patients with high cardiovascular risk. Combining coenzyme Q10 (10 mg, t.i.d., p.o.) with trimetazidine (35 mg, b.i.d., p.o.) is recommended for oral administration, and creatine phosphate is suitable for intravenous treatment (2.0 g, q.d., i.v. drip).

#### 2.4 Mechanical life support

Application of mechanical circulatory support systems, including IABP and ECMO, should be considered for patients with cardiogenic shock caused by highly suspected fulminant myocarditis or AMI. The main principles are: (i) control the cardiovascular risks at an early stage; (ii) stay alert to signs of unstable hemodynamics; (iii) implant mechanical life support as early as possible when a cardiogenic shock occurs. Patients who advance into multiple organ disorder syndrome (MODS) are difficult to save with the life support.

##### *2.4.1 Indications for COVID-19 patient to implant IABP*

(1) AMI with unstable hemodynamics, BP $< 90/60$ mmHg.

(2) NT-proBNP shows abnormally higher beyond the normal range, patients manifested refractory heart failure.

(3) Highly suspected fulminant myocarditis.

(4) Septic shock.

##### *2.4.2 Indications for COVID-19 patient to implant ECMO*

Age $< 70$ years old, without known severe brain injury, unrecoverable heart, or uncontrollable hemorrhage. If the mechanical ventilation settings for patients with COVID-19 and ARDS have been optimized (low tidal volumes $<$ 6 mL/kg (ideal body weight) and airway plateau pressure $< 30$ $cmH_2O$, PEEP $\geqslant 10$ $cmH_2O$, $FiO_2$ $\geqslant 0.8$), the prone positioning has been applied for at least 12 h per day. If the patient's hypoxia is still not relieved, ECMO should be considered as early as possible.

(1) $PaO_2/FiO_2 < 50$ mmHg more than 3 h.

(2) $PaO_2/FiO_2 < 80$ mmHg more than 6 h.

(3) $FiO_2 = 1.0$, $PaO_2/FiO_2 < 100$ mmHg.

(4) Arterial blood gas pH $< 7.25$, $PaCO_2 > 60$ mmHg more than 6 h, RR $> 35$.

(5) RR $> 35$, pH $< 7.2$, Pplat $> 30$ $cmH_2O$.

If the patient's cardiac function is well maintained, we prefer VV-ECMO, unless there was a concomitant circulatory failure. IABP combined with VA-ECMO could be applied to the patient who has left ventricular dysfunction or refractory cardiogenic shock.

### 3 Renal function support

Recent studies have shown that the kidney may be a target organ of COVID-19 infection [11]. Renal supportive therapy is important in maintaining the balance of water, electrolyte, and acid–base in the body [12]. Inflammatory factor storm is a major pathophysiological mechanism in the pathogenesis of severe and critical cases of COVID-19 [11]. The effective elimination of inflammatory factors through the blood purification program could avoid multi-organ functional damage, improve the cure rate, and reduce mortality [13–15]. In the clinical course of patients with severe- and critical-type COVID-19, we propose the following recommendations for kidney support therapy and blood purification therapy.

#### 3.1 General treatment of renal function support

##### *3.1.1 Closely monitor renal function, electrolyte, and volume load*

For patients with normal renal function, renal function and electrolyte (including natrium, chlorine, potassium, calcium, and magnesium) levels should be checked every 3–5 days. For patients combined with renal insufficiency, the examination frequency must be increased and urinalysis must be monitored. The liquid intake and output volume must be recorded. Central venous pressure (CVP) and cardiac index (CI) must be monitored in critically ill patients to evaluate volume load accurately.

*3.1.2 Reduce the use of nephrotoxic drugs*

Patients with severe- and critical-type COVID-19 usually have multiple system damages. Thus, nephrotoxic drugs should be avoided. For example, vancomycin and teicoplanin should be avoided in patients with Gram-positive cocci infection. For patients combined with renal insufficiency, drug contraindications should be noticed. Dose of some drugs needs to be adjusted according to eGFR. Dose adjustment for arbidol and lopinavir/ritonavir is not needed in patients with renal insufficiency.

*3.1.3 Early detection of acute kidney injury (AKI) and continuous renal replacement therapy (CRRT) if necessary*

Studies have confirmed that 10%–15% of patients with severe- and critical-type COVID-19 have AKI. The main reasons are renal perfusion insufficiency resulting from hypotension or circulatory failure, applications of nephrotoxic medications, direct and indirect renal damage caused by virus infection, and renal injury caused by failure of vital organs, such as heart and lung. If renal dysfunction occurs, water electrolytes and acid–base balance should be closely, monitored for early detection of AKI. In case of AKI, the pathogenic factors should be actively found and removed, and CRRT treatment should be given when necessary.

### 3.2 CRRT treatment in patients with COVID-19

*3.2.1 Indications for CRRT in patients with COVID-19*

(1) Patients undergoing maintenance hemodialysis were suspected, confirmed, or clinically diagnosed with COVID-19.

(2) Patients complicated with AKI, which results in severe electrolyte and acid–base disturbance or volume overload, need blood purification treatment.

(3) Patients with the complications of MODS, sepsis, severe inflammatory reaction syndrome, macrophage activation syndrome.

(4) Patients with severe disorders of water, electrolyte, and acid–base balance caused by other reasons, which are difficult to correct with routine treatment.

*3.2.2 Contraindications of CRRT in COVID-19 patients*

There is no absolute contraindication of CRRT treatment in patients with COVID-19. However, CRRT should be used discreetly when hypotension is difficult to correct or proper vascular access is hard to establish.

*3.2.3 CRRT procedure for uremic patients with COVID-19*

(1) Continuous veno-venous hemofiltration (CVVH)/continuous veno-venous hemodiafiltration (CVVHDF) should be taken as the routine hemodialysis pattern, with a therapeutic dose of 4–6 L/h. Each therapeutic time lasts 4–6 h, with a treatment frequency of twice to three times a week.

(2) Anticoagulants should be chosen according to the patient's condition. Heparin or low-molecular-weight heparin is recommended in patients without active bleeding and hemorrhage proneness. Citrate is suggested for patients with heparin intolerance, thrombocytopenia, and bleeding tendency.

*3.2.4 CRRT procedure for non-uremic patients with COVID-19*

(1) Practice patterns

(i) The baseline mode is CVVH, and the therapeutic dose is 20–75 mL/kg/h. Patients' condition should be considered when choosing pre-dilution or post-dilution. CVVHDF is beneficial for patients suffering from serious electrolyte and acid–base imbalance or with high levels of small molecular weight metabolite (such as hypermetabolism of protein).

(ii) For patients with high inflammatory response, plasmapheresis, and filtration, plasma filtration absorption and protein A immune absorption are available for scavenging inflammatory cytokines and protecting important organs. The definition of hyper inflammatory response is serum IL-6 $>$ 42 pg/mL, IL-2R $>$ 900 U/mL, tumor necrosis factor α (TNF-α) $>$ 48.8 pg/mL, and/or high-sensitivity CRP $>$ 120 mg/L.

(2) Ultrafiltration

Patient's volume load should be comprehensively evaluated according to individual inflow and outflow, CVP, CI, and so on. Patients with severe- and critical-type COVID-19 have poor tolerance to hypotension because of long-term hypoxia. For patients with unstable vital signs, ultrafiltration should be performed slowly and continuously to avoid the occurrence of hypotension as much as possible. Ultrafiltration curve can be adopted when necessary. The ultrafiltration speed at the initial stage is low, gradually increases, and finally gradually reduces until the end of CRRT treatment. In patients with severe edema, hydrothorax, or ascites and hypoproteinemia, albumin (ALB) and other colloidal substances can be supplied during the therapy.

(3) Anticoagulation

Heparin or low-molecular-weight heparin is routinely recommended for anticoagulation. The specific anticoagulant method should be comprehensively judged according to liver function, platelet count, ALB, blood lipid level, and the general condition of the patients. The contraindications of various anticoagulants should be understood. Anticoagulation should not only ensure the therapeutic effect but also avoid serious bleeding or coagulation complications.

Precautions are as follows: (i) citric acid and argatroban are contraindicated in patients with severe liver dysfunction; (ii) citrate anticoagulation is contraindicated in patients with severe hypocalcemia or acidosis; (iii) heparin is contraindicated in patients with heparin-related thrombocytopenia or heparin allergy; (iv) patients with thrombocytopenia ( $< 30 \times 10^9$), severe coagulopathy, clotting factor deficiency, or bleeding tendency may not need anticoagulants.

##### *3.2.5 Indications of ceasing CRRT*

(1) If patients with AKI are in remission and renal function is gradually restored after removing triggers, CRRT interval could be gradually lengthened until the cessation. If COVID-19 is cured but kidney function is still compromised, peritoneal dialysis or maintenance hemodialysis can be considered.

(2) For patients subjected to CRRT because of hyper inflammatory response or severe imbalance of water, electrolyte, and acid–base, CRRT is considered to be discontinued when related inflammatory parameters are reduced to normal or near normal range, multiple organ failure is controlled, respiratory function is improved, and their volume is in good control.

### 4 Liver function support

#### 4.1 Manifestation of liver injury in patients with COVID-19

Liver injury is common in patients with COVID-19, especially in those severely or critically ill patients [2,16–18]. Previous investigations reported that the pathological findings of COVID-19 revealed moderate microvascular steatosis and mild lobular and portal activity in the liver, reflecting that the liver injury of COVID-19 is probably caused by ischemia, hypoxia, and inflammatory factors. The clinical characteristics of liver injury of COVID-19 are as follows.

##### *4.1.1 Levels of injury biomarkers of hepatocytes*

Levels of injury biomarkers of hepatocytes, including alanine aminotransferase, aspartate aminotransferase (AST), and lactate dehydrogenase, are usually elevated.

##### *4.1.2 Hypoalbuminemia*

ALB is synthesized in the liver and initially released into blood.

##### *4.1.3 Coagulopathy*

Coagulopathy is manifested as prolonged prothrombin time (PT), decreased prothrombin activity, and elevated international normalized ratio (INR) in the end stage of severe-type COVID-19.

##### *4.1.4 Bile duct injury*

In some patients with COVID-19, bile duct injury markers, such as alkaline phosphatase (ALP) and $\gamma$ glutamyltransferase ($\gamma$-GT), are increased, but the elevation of bilirubin levels is rare (found in some patients with critical-type COVID-19).

##### *4.1.5 Severe liver injury*

Isolated severe liver injury is rare and usually accompanied by shock, respiratory failure, cardiac insufficiency, and/or renal insufficiency in patients with critical-type COVID-19.

In addition, after liver injury, the synthetic function of liver is damaged. Consequently, the tissue repair response of human body to viral infection is impaired. Thus, the liver function of patients should be monitored during the treatment of COVID-19.

#### 4.2 Key points of monitoring liver function in patients with COVID-19

##### *4.2.1 Medical history*

The medical history of patients with COVID-19 must be taken to confirm whether they have a history of chronic liver disease and identify whether they have taken drugs with liver injury potential, including arbidol, lopinavir/

ritonavir tablets, moxifloxacin, acetaminophen, and other drugs commonly used in patients with COVID-19.

*4.2.2 Maximizing the existing results of radiological examination*

In the plain CT scan of the chest, cirrhosis may be considered when the radiological features of the liver show that the liver lobe is reduced or out of proportion, the liver fissure is widened, the portal area is enlarged, the liver edge is irregular, and the spleen is enlarged. The ratio of the CT value of the liver and spleen can be used to evaluate the existence of liver steatosis and its severity.

*4.2.3 Interpretation of the biochemical profile of liver function*

ALT and AST are commonly used markers of hepatic injury, whereas ALP and γ-GT are commonly used as markers of bile duct injury. Common indices in evaluating liver synthetic function, including ALB, PT, bilirubin, pre-ALB, blood cholesterol, and cholinesterase levels, not only can reflect the severity of liver injury but also can assess the regeneration capacity of the liver. In addition, the increase of AST can also be caused by myocardial injury; the decrease of ALB in patients with severe-type COVID-19 is related to the factors of exhaustion of the ALB storage, insufficient nutrition support, and decreased synthesis of the liver. When disseminated intravascular coagulation occurs in critically ill patients with COVID-19, PT is also significantly prolonged. The comprehensive evaluation of the above index should be patient specific.

*4.2.4 Using the ultrasonic scan of the liver*

The ultrasonic scan of the liver is commonly used to evaluate cirrhosis, steatosis, and tumorigenic lesion in the liver. In consideration that hypercoagulable state and thrombus activity are common in patients with critical-type COVID-19, liver color Doppler ultrasound can be used to assess the flow rate of portal vein and hepatic artery and the presence of thrombus.

*4.2.5 Etiological detection*

For COVID-19 patients with liver injury, blood test should include a complete set of viral hepatitis antibody. HBV-DNA or HCV-RNA nucleic acid test should be completed for those with positive HBV or HCV antibody, and corresponding antiviral treatment should be performed for those patients with high level of hepatitis virus titer in the blood.

### 4.3 Treatment of liver injury in COVID-19

*4.3.1 Symptomatic treatment*

Symptomatic treatment of liver injury is also termed as liver protection therapy. In essence, liver protection therapy can be divided into two categories: anti-inflammatory therapy and supplementary therapy. Several types of these therapeutic drugs have dual functions mentioned above.

(1) Anti-inflammatory treatment refers to reducing inflammatory factors to hepatocytes and clearing some inflammatory factors. Specific drugs include glycyrrhizic acid drugs, reduced glutathione, ω-3 unsaturated fatty acid, glucose-insulin-potassium solution, N-acetylcysteine, polyene phosphatidylcholine, and so on. The first three drugs can also reduce inflammation in the whole body and have no side effects of steroidal anti-inflammatory drugs.

(2) Supplementary therapy supplements the products of liver synthesis and reduces the synthetic and metabolic load of the damaged liver. It includes ALB, branched chain amino acids (compound amino acid injection), ornithine aspartate, vitamins, and coenzymes (inosine, VitC, cytochrome C, coenzyme A), and so on.

A combination of two to three drugs mentioned above is usually used to treat liver injury. In the majority of patients with critical-type COVID-19, the level of ALB is significantly reduced. We recommended to supply ALB to ensure nutritional support (especially amino acid supply) and maintain the level of circulating ALB above 35 g/L.

*4.3.2 Etiological treatment*

If the pathogenesis of liver injury persists, the therapeutic effect of liver protection therapy is limited. The pathogenesis of liver injury in patients with COVID-19 may include the attack of inflammatory factors to the liver, ischemia- and hypoxia-related liver injury, drug-induced liver injury, virus infection of the liver, and the negative influence of underlying liver diseases. In the treatment, we recommend to treat the cause of liver injury actively, which include:

(1) Active respiratory support and/or circulatory support to treat the ischemia and hypoxia in patients with critical-type COVID-19.

(2) Anti-inflammatory therapy and extracorporeal blood purification techniques for the treatment of COVID-19 reduce the inflammatory response, and the elimination of inflammatory factors can effectively reduce the inflammatory liver injury.

(3) Arbidol, lopinavir/ritonavir tablets, and moxifloxacin may increase liver enzymes or even cause severe liver injury. At present, evidence showing that arbidol and lopinavir/ritonavir have antiviral effects on SARS-CoV-2 is currently lacking. Thus, drugs with potential hepatoxicity should be stopped or replaced when patients with COVID-19 have liver damage. If patients have chronic liver disease, we recommended to reduce the use of drugs with potential hepatoxicity.

### 5 Hematological system function support

#### 5.1 COVID-19 with febrile neutropenia (neutrophil count $\leqslant 0.5 \times 10^9$/L)

##### *5.1.1 Medical history*

Doctors should record the medical history of patients with COVID-19 to check if they are complicated with any hematological malignancies. If the patients have a history of leukemia, lymphoma, or multiple myeloma, doctors should distinguish the reason of febrile neutropenia to see if it is in the state of myelosuppression after chemotherapy or in the state of uncontrolled primary disease. The complete blood count, CRP, PCT, and serum cytokine profile including IL-1β, IL-2R, IL-6, IL-8, IL-10, TNF-α, and ferritin should be monitored regularly. Hematological specialists should be consulted if possible.

##### *5.1.2 Blood culture collection*

For patients with persistent fever and temperature greater than 38.0 °C, blood culture must be obtained from both upper arms. For patients with peripherally inserted central catheter (PICC), three sets of blood cultures with a culture of the PICC tip should be performed simultaneously. Next-generation sequencing for pathogen is also recommended to obtain unbiased potential pathogen. The appropriated antibiotics should be selected according to the drug sensitivity results. The indication for blood culture collection is when the patient had persistent fever before the use of antibiotics. Patients should receive multiple blood culture collection if complicated with persistent high fever or chilly or in the state of changing the antibiotics.

##### *5.1.3 Use of antibiotics*

If febrile neutropenia exists, intravenous antibiotics should be immediately used without waiting for the results of blood culture. The first choice is carbapenems or cephalosporins with β-lactamases. If the fever persists after use of the antibiotics, the antibiotics against Gram-positive cocci should be considered. Invasive fungal infection should also be suspected with G/GM tests and typical CT findings. If necessary, hematological specialists must be consulted to adjust the appropriate antibiotics.

##### *5.1.4 Clinical fluid replacement*

The fluid should be supplemented if the patients had sweating, dry mouth, oliguria, poor skin elasticity, and high urinometry. The fluid replacement should be used if nonsteroidal anti-inflammatory drugs are applied: first expansion, after adjustment of positive electrolyte and acid–base balance; use of crystal after the use of limbs. Rehydration rate: first fast and then slow. Usually 60 drops per minute. The speed for rescue of shock should be faster. The potassium should be supplemented if the urine is greater than 40 mL/h. For moderate dehydration, 100 mL sodium bicarbonate should be supplemented for every 800 mL normal saline.

##### *5.1.5 Use of G-CSF*

If the patient is in the state of myelosuppression after chemotherapy, the G-CSF is recommended to be used to improve the recovery of WBC. G-CSF i.p. at 100–300 μg should be applied and adjusted according to the WBC count.

#### 5.2 COVID-19 with disseminated intravascular coagulation

##### *5.2.1 Bleeding risk evaluation*

Doctors should check for hematemesis, black stool (melena), hemoptysis, gross hematuria, and broad-skin ecchymosis. For patients with these conditions, their platelet and fresh frozen plasma (FFP) should be immediately supplemented. The red blood cells should also be supplemented according to the condition of blood loss.

##### *5.2.2 Fibrinogen supplement*

The replacement of fibrinogen (Fbg) should be suggested if the Fbg level is less than 0.5 g/L (first 60 mg/kg with 20 mg/kg maintenance, 0.5 g/bottle). If fibrinogen is not available, cryoprecipitate should be considered. One unit of cryoprecipitate contains approximately 75 mg fibrinogen.

##### *5.2.3 Platelet infusion*

The platelet should be maintained at a level above

$30\times10^9$–$50\times10^9$/L; if the patient is using heparin, the $50\times10^9$/L platelet count should be maintained.

*5.2.4 Red blood cell infusion*

Red blood cells should be replaced if the hemoglobin level is less than 60 g/L, or 80 g/L hemoglobin level should be retained if the patient has chest palpitations or active hemorrhage. If the patient has no severe tendency of hemorrhage or no invasive examination, or the prolong of APTT is not twice greater than normal values and/or INR is less than 2, the prophylactic transfusion of blood products is generally not advised.

*5.2.5 Microcirculation failure or micro-angiopathic hemolytic anemia*

Microcirculation failure or micro-angiopathic hemolytic anemia should be carefully treated; the doctors should carefully observe the patient's urine volume, skin elasticity, conscious state, vital sign, yellow sclera, and so on. Acidosis should be corrected immediately if existing.

#### 5.3 COVID-19 with acute hemolysis

The clinical symptoms include jaundice, anemia, shock, and renal failure.

*5.3.1 Recommendation*

Prednisolone at 1–1.5 mg/kg should be used (10 mg dexamethasone if any emergency). Consideration must be given to the treatment of COVID-19 depending on the dosage and time duration of the corticosteroid. Hematological specialists must be consulted if any was unconfirmed.

*5.3.2 Alkalization of urine*

Intravenous infusion of 125 mL sodium bicarbonate should be given, and this procedure can be repeated every 24 h if necessary.

*5.3.3 Fluid replacement*

The speed of fluid replacement should be slow if severe anemia exists.

*5.3.4 Immunoglobulin therapy*

If the diagnosis of hemolytic anemia is confirmed, the intravenous immunoglobulin therapy is suggested: 20 g once daily for 3 days, which is beneficial for COVID-19 and acute hemolysis.

*5.3.5 Transfusion*

Washed red blood cells instead of suspended red blood cells should be used in patients with hemolysis.

*5.3.6 Patients with PNH*

Low-molecular-weight dextran, 500–1000 mL.

*5.3.7 Hematopoietic raw materials should be simultaneously supplemented in patients with hemolytic anemia*

Oral intake of 500 μg vitamin $B_{12}$ every day, three times daily and 10 mg folic acid every day, once daily.

#### 5.4 COVID-19 patients with immune thrombocytopenic purpura (ITP)

Doctors should evaluate if the patients have any active hemorrhage or are at high risk of hemorrhage. If the previous ITP is confirmed, the platelet replacement is not generally suggested if the patients have no tendency to bleed. If the diagnosis of ITP is not completely confirmed and the patients have active hemorrhage or are at high risk of hemorrhage, the prophylactic platelet replacement should be given especially for patients with platelet count less than $10 \times 10^9$/L. It is generally safe for patients with platelet count greater than $80 \times 10^9$/L.

Intravenous immunoglobulin plus oral eltrombopag tablets should be suggested for patients with ITP. The detailed usage is as follows: intravenous 20 g of immunoglobulin once daily for consecutive 3 days, which is beneficial for both COVID-19 and ITP. Oral intake of 50 mg eltrombopag tablets once a day (please consult hematological specialists if unsure) is recommended. The dosage of the drug should be adjusted accordingly after recovery of platelet count. The use of corticosteroids should be cautious depending on the disease condition of COVID-19.

#### 5.5 Transfusion of blood products in patients with severe/critical-type COVID-19

*5.5.1 Platelet transfusion therapy*

Platelet transfusion therapy is recommended to maintain the platelet count to following threshold range on the following conditions:

(1) If non-fatal bleeding occurs, the platelet count should be maintained at least greater than $30 \times 10^9$/L.

(2) If sever bleeding occurs, the platelet count should be maintained at least greater than 50 $\times$ $10^9$/L.

(3) If fatal bleeding occurs, such as multiple trauma, traumatic brain injury, and/or spontaneous intracranial hemorrhage, the platelet count should be maintained at least greater than 100 $\times$ $10^9$/L.

*5.5.2 Irradiated blood products*

The irradiated blood products are used to deplete the lymphocytes to reduce the risk of transfusion associated graft-versus-host disease (GVHD): if the patients have received any chemotherapy regimens containing rituximab and/or fludarabine, the irradiated blood products should be used. If irradiated blood products are not available in the department of blood transfusion, the blood product with filtered white blood cells should be used and a special filter for transfusion should be added.

*5.5.3 Blood products with filtered white blood cells*

These products have filtered the white blood cells in the blood products to reduce the risk of transfusion-associated GVHD. Blood products with filtered white blood cells must be used as much as possible for patients with hematological malignancies. If it is not available, the ordinary blood products can be used. If only the ordinary blood products are sent back to the ward even doctors applied for filtered blood products, they should be cautiously used for avoiding any nonessential waste.

*5.5.4 Washed red blood cells*

This blood product is indicated to patients with hemolysis.

*5.5.5 Blood transfusion in febrile patients*

The platelet should be infused within 1 h after being received from the department of transfusion. Body temperature is not a strict contraindication for platelet transfusion. The same condition can be applied for patients who need emergent hemostasis or emergent red blood cell transfusion. If the transfusion is not an emergent treatment, physical hypothermy, non-steroidal anti-inflammatory drug, or corticosteroid can be first used to control the body temperature. Then, the transfusion treatment can be proceeded when the body temperature is less than 38 °C. All blood transfusion products should be kept on 4 °C.

*5.5.6 Fresh frozen plasma*

The initial dose of FFP should be 10–15 mL/kg with a maintenance dose of 5–10 mL/kg. The most coagulation factor in peripheral blood can be elevated to 25%–30% when the total 10–20 mL/kg FFP is infused. Every 1 unit of cryoprecipitate is prepared by 400 mL whole blood with the final volume of 25 $\pm$ 5 mL. One unit of cryoprecipitate contains approximately $\geqslant$ 80 IU factor VIII, 150 mg fibrinogen, von Willebrand factor (vWF), factor XIII, and fibronectin from the FFP. The dosage of 1–1.5 U/10 kg cryoprecipitate is suggested and is mainly applied in patients with deficiency of factor VIII and vWF or in hemorrhagic patients with low or absent fibronectin and/or fibrinogen.

### 6 Anti-thrombotic therapy

#### 6.1 Risk assessment of venous thromboembolism (VTE) in patients with COVID-19

Deep venous thrombosis (DVT) is mainly caused by secondary risk factors, such as severe infection, hypercoagulable state, prolonged bed rest ( $>$ 48 h), obesity, older age, history of atrial fibrillation, and hypertension. The following elements affecting the blood status of patients with COVID-19 can be observed.

(1) Most patients with COVID-19 treated in our hospital have fever, occasional diarrhea, and vomiting, resulting in serious bodily fluid loss and hemoconcentration.

(2) In severe and critical patients, inflammatory factors are released, D-dimer is generally abnormally increased, and the blood is in a state of hypercoagulability.

(3) Patients with liver function damage, decreased ALB, decreased colloidal osmotic pressure, fluid exudation, and blood state also show changes.

(4) Dyspnea, fatigue and long-term bed treatment, slow limb venous blood reflux, and blood stagnation can be observed. In addition, the elderly, a history of VTE, other underlying diseases (tumor, obesity, diabetes, hypertension, etc.), central venous catheterization, IABP, ECMO, and other procedures increase the risk to VTE of patients with COVID-19.

#### 6.2 Clinical manifestations and early diagnosis of VTE and pulmonary embolism (PE) in patients with COVID-19

*6.2.1 DVT*

Acute lower limb deep venous thrombosis should be considered in patients who have sudden swelling, pain, and elevated skin temperature of one limb, especially one side of the limb (calf or ankle area). Color Doppler ultrasound of both lower limbs is the first choice and the most common examination to find thrombosis. Phlebography is the gold standard for the diagnosis of this disease. The diagnosis of DVT is mainly based on clinical symptoms, elevated level of D-dimer, and positive findings of Doppler ultrasound in lower extremity veins.

COVID-19 patients with no typical symptoms and slightly elevated level of D-dimer can be temporarily observed because some DVT patients may have no typical symptoms while the basic D-dimer level of COVID-19 patients is increased. Asymptomatic patients whose D-dimer level is significantly increased or those with treated systemic infection nonetheless dynamic increase of D-dimer level should be diagnosed by lower limb venous Doppler ultrasound. Meanwhile, for severe and critical COVID-19 patients with suspected DVT, Doppler ultrasonography of lower extremity veins must be performed at the bedside.

##### *6.2.2 PE*

In consideration that patients with COVID-19 may have dyspnea and fever, PE should be considered if dyspnea aggravates, hypoxemia is serious, and blood pressure drops abnormally. Patients with highly suspected PE must urgently check D-dimer and CTPA (computed tomographic pulmonary angiography). Other possible examinations include electrocardiogram (typical $S_IQ_{III}T_{III}$, sinus tachycardia, right bundle branch block, ST-T changes), echocardiography, and so on.

#### 6.3 Early prevention of VTE and PE in patients with COVID-19

Combined with the above risk factors and clinical symptoms, precautions against VTE and PE should be taken by patients with COVID-19 regardless of severity. Patients with mild COVID-19 are encouraged to do more exercise and walk out of bed. For patients with severe- and critical-type COVID-19, risk assessment scales such as Welles's score can be used for evaluation to prevent VTE. Low-risk patients are advised to strengthen exercise. Medium- and high-risk patients can be subcutaneously injected with a half-dose of low-molecular-weight heparin once every 12 h or subcutaneously injected with one branch of low-molecular-weight heparin once a day for prevention. In addition, physical prevention methods, such as artificial-assisted exercise, anti-venous-embolism massage apparatus, and elastic socks, can also be applied for early prevention. For severe or critical patients who cannot be transferred to CTPA, intensive anticoagulation therapy is feasible in the absence of anticoagulation taboo.

#### 6.4 Anti-thrombotic therapy in patients with COVID-19

##### *6.4.1 DVT*

For patients who have been clearly examined with DVT, treatment methods are as follows.

(1) Basic treatment measures of patients with COVID-19 include fluid replacement, correction of water and electrolyte balance, protein supplementation, and anti-inflammation, which can ameliorate the blood hypercoagulable state to a certain extent as well.

(2) Patients with acute DVT should exercise less, elevate the affected limb and forbid massage to prevent embolism from falling off and blocking pulmonary vessels to form PE.

(3) Patients with mild COVID-19 should be given oral anticoagulants or low-molecular-weight heparin and warfarin. The preferred treatment dose is rivaroxaban 20 mg once a day, but it is likely to increase mainly through the CYP3A drug metabolism of blood drug concentration, during the treatment of combination with lopinavir and ritonavir. One branch of low-molecular-weight heparin can be subcutaneously injected once every 12 h and warfarin 2.5–3 mg per day at the beginning. The blood coagulation function is dynamically monitored during medication, and the dose is adjusted according to the INR to maintain 2.0–3.0.

(4) For patients with severe- and critical-type COVID-19, one branch of low-molecular-weight heparin is the first choice for anticoagulation therapy, which should be subcutaneously injected once every 12 h. The dosage should be adjusted according to body weight. After 3–5 days, the patients are treated with new oral anticoagulants (such as rivaroxaban, 15 mg, twice a day, for the first 3 weeks) or warfarin (the initial dose is 2.5 mg, once a day, orally). Low-molecular-weight heparin is discontinued after the INR reaches the standard (2–3) and stabilizes for 24 h.

(5) For special patients with venerable age ( $\geqslant$ 80 years old), low body weight ( $\leqslant$ 50 kg), and mild-to-moderate renal insufficiency (creatinine clearance rate is 30–80 mL/min), preventive anticoagulant therapy dose (1/2 dosage) can be used. For patients with severe renal insufficiency (creatinine clearance $\leqslant$ 30 mL/min) but without hemodialysis or bedside ultrafiltration, unfractionated heparin is recommended. For gravida, the anticoagulant regimen of low-molecular-weight heparin is recommended.

(6) The routine use of inferior vena cava filter is not recommended for patients with deep venous thrombosis treated with anticoagulation alone, and implantation of inferior vena cava filter is recommended for those who have contraindications or complications in anticoagulation therapy or PE in adequate anticoagulation therapy.

(7) During anticoagulant therapy, dynamic monitoring of blood routine and D-dimer level and bleeding must be performed, and anticoagulant therapy must be adjusted or terminated in time. The use of heparin drugs may cause heparin-induced thrombocytopenia (HIT). For patients with HIT during the use of heparin, it is advised to be replaced by other anticoagulants, such as argatroban, bivalirudin, rivaroxaban, and fondaparinux sodium.

#### *6.4.2 PE*

The treatments are mainly anticoagulation and thrombolysis. A small number of emergency patients who are not suitable for drug treatment can use intervention or surgery.

(1) Thrombolytic therapy: The thrombolytic drugs currently used can be divided into two categories: specific binding fibrinolytic agents and non-specific binding fibrinolytic thrombolytic agents. The former commonly includes recombinant tissue-type plasminogen activator rt-PA and r-PA, whereas the latter are streptokinase and urokinase. Common dose: rPA: 10 MU of this product is dissolved in 10 mL injection water each time, slowly pushed in 2–5 min. Another 10 MU can be used if necessary. The interval between the two times is about 30 min. A separate intravenous pathway should be used during injection and should not be mixed with other drugs. It also should not be given in common with other drugs. rt-PA: 50–100 mg is given intravenously and lasts for 2–4 h. The first dose of UK (4000 U/kg) is injected intravenously within 30 min, followed by drip of 600 000–1.2 million unit within 2–4 h.

(2) Anticoagulant therapy: the same as deep vein thrombosis.

(3) Special patients need intervention or surgery must be performed in a negative pressure operating room.

### Conclusions

Early identification and active intervention are the bases for reducing the mortality of patients with critical-type COVID-19. Multi-disciplinary and individualized treatment plays a key role in reducing the mortality of patients with critical-type COVID-19. In general, early warning, timely intervention, individualized management, and multi-disciplinary organ support treatment are highly necessary to treat patients with severe- and critical-type COVID-19. We hope that our experience in diagnosis and treatment can provide a reference and optimal treatment strategy for countries worldwide suffering from COVID-19 to reduce mortality of patients with COVID-19.

### Members of Multidisciplinary Team for COVID-19 in Optical Valley Branch of Tongji Hospital (Sorted by last name)

**Peng Chen** (1 Department of Cardiology, Department of Internal Medicine and Genetic Diagnosis Center, Tongji Hospital, Tongji Medical College, Huazhong University of Science and Technology; 2 Hubei Key Laboratory of Genetics and Molecular Mechanism of Cardiological Disorders, Wuhan 430030, China)

**Xiaoping Chen** (1 Hepatic Surgery Center, 2 Hubei Key Laboratory of HPB Diseases, Tongji Hospital, Tongji Medical College, Huazhong University of Science and Technology, Wuhan 430030, China)

**Zeyang Ding** (1 Hepatic Surgery Center, 2 Hubei Key Laboratory of HPB Diseases, Tongji Hospital, Tongji Medical College, Huazhong University of Science and Technology, Wuhan 430030, China)

**Shuiming Guo** (Department of Nephrology, Department of Internal Medicine, Tongji Hospital, Tongji Medical College, Huazhong University of Science and Technology, Wuhan 430030, China)

**Xiaomei Guo** (1 Department of Cardiology, Department of Internal Medicine and Genetic Diagnosis Center, Tongji Hospital, Tongji Medical College, Huazhong University of Science and Technology; 2 Hubei Key Laboratory of Genetics and Molecular Mechanism of Cardiological Disorders, Wuhan 430030, China)

**Fan He** (Department of Nephrology, Department of Internal Medicine, Tongji Hospital, Tongji Medical College, Huazhong University of Science and Technology, Wuhan 430030, China)

**Rui Li** (Department of Gastroenterology, the First Affiliated Hospital of Soochow University, Suzhou 215006, China)

**Shengqing Li** (Department of Pulmonary and Critical Care Medicine, Huashan Hospital, Fudan University, Shanghai 200040, China)

**Yong Li** (Department of Respiratory and Critical Care Medicine, Department of Internal Medicine, Tongji Hospital, Tongji Medical College, Huazhong University of Science and Technology, Wuhan 430030, China)

**Xiaoning Wan** (1 Department of Cardiology, Department of Internal Medicine and Genetic Diagnosis Center, Tongji Hospital, Tongji Medical College, Huazhong University of Science and Technology; 2 Hubei Key Laboratory of Genetics and Molecular Mechanism of Cardiological Disorders, Wuhan 430030, China)

**Daowen Wang** (1 Department of Cardiology, Department of Internal Medicine and Genetic Diagnosis Center, Tongji Hospital, Tongji Medical College, Huazhong University of Science and Technology; 2 Hubei Key Laboratory of Genetics and Molecular Mechanism of Cardiological Disorders, Wuhan 430030, China)

**Hui Wang** (Department of Gynecologic Oncology, Tongji Hospital, Tongji Medical College, Huazhong University of Science and Technology, Wuhan 430030, China)

**Luyun Wang** (1 Department of Cardiology, Department of Internal Medicine and Genetic Diagnosis Center, Tongji Hospital, Tongji Medical College, Huazhong University of Science and Technology; 2 Hubei Key Laboratory of Genetics and Molecular Mechanism of Cardiological Disorders, Wuhan 430030, China)

**Jia Wei** (Department of Hematology, Tongji Hospital, Tongji Medical College, Huazhong University of Science and Technology, Wuhan 430030, China)

**Gang Xu** (Department of Nephrology, Department of Internal Medicine,

Tongji Hospital, Tongji Medical College, Huazhong University of Science and Technology, Wuhan 430030, China)

**Zhenyu Yin** (Department of Hepatobiliary Surgery, Zhongshan Hospital, Xiamen University, Xiamen 361004, China)

**Wenkui Yu** (Department of Critical Care Medicine, Gulou Hospital, Nanjing University, Nanjing 210008, China)

**Bixiang Zhang** (1 Hepatic Surgery Center, 2 Hubei Key Laboratory of HPB Diseases, Tongji Hospital, Tongji Medical College, Huazhong University of Science and Technology, Wuhan 430030, China)

**Binhao Zhang** (1 Hepatic Surgery Center, 2 Hubei Key Laboratory of HPB Diseases, Tongji Hospital, Tongji Medical College, Huazhong University of Science and Technology, Wuhan 430030, China)

**Jianping Zhao** (Department of Respiratory and Critical Care Medicine, Tongji Hospital, Tongji Medical College, Huazhong University of Science and Technology, Wuhan 430030, China)

**Jianfeng Zhou** (Department of Hematology, Tongji Hospital, Tongji Medical College, Huazhong University of Science and Technology, Wuhan 430030, China)

**Ning Zhou** (1 Department of Cardiology, Department of Internal Medicine and Genetic Diagnosis Center, Tongji Hospital, Tongji Medical College, Huazhong University of Science and Technology; 2 Hubei Key Laboratory of Genetics and Molecular Mechanism of Cardiological Disorders, Wuhan 430030, China)

**Wei Zhu** (Department of Emergency and Critical Care, Tongji Hospital, Tongji Medical College, Huazhong University of Science and Technology, Wuhan 430030, China)

### Compliance with ethics guidelines

The authors declare that they have no conflicts of interest. This manuscript does not involve a research protocol requiring approval by the relevant institutional review board or ethics committee.

**Open Access** This article is licensed under a Creative Commons Attribution 4.0 International License, which permits use, sharing, adaptation, distribution and reproduction in any medium or format, as long as you give appropriate credit to the original author(s) and the source, provide a link to the Creative Commons licence, and indicate if changes were made.

The images or other third party material in this article are included in the article's Creative Commons licence, unless indicated otherwise in a credit line to the material. If material is not included in the article's Creative Commons licence and your intended use is not permitted by statutory regulation or exceeds the permitted use, you will need to obtain permission directly from the copyright holder.

To view a copy of this licence, visit https://creativecommons.org/licenses/by/4.0/.
